# Contact-Dependent Growth Inhibition Proteins in *Acinetobacter baylyi* ADP1

**DOI:** 10.1007/s00284-018-1540-y

**Published:** 2018-07-17

**Authors:** Eliana De Gregorio, Eliana Pia Esposito, Raffaele Zarrilli, Pier Paolo Di Nocera

**Affiliations:** 1 Dipartimento di Medicina Molecolare e Biotecnologie Mediche, Università Federico II, Via Sergio Pansini 5, 80131 Naples, Italy; 2 Dipartimento di Sanità Pubblica, Università Federico II, Via Sergio Pansini 5, 80131 Naples, Italy

## Abstract

Bacterial contact-dependent growth inhibition (CDI) systems are two-partner secretion systems in which toxic CdiA proteins are exported on the outer membrane by cognate transporter CdiB proteins. Upon binding to specific receptors, the C-terminal toxic (CT) domain, detached from CdiA, is delivered to neighbouring cells. Contacts inhibit the growth of not-self-bacteria, lacking immunity proteins co-expressed with CdiA, but promote cooperative behaviours in “self” bacteria, favouring the formation of biofilm structures. The *Acinetobacter baylyi* ADP1 strain features two CdiA, which differ significantly in size and have different CT domains. Homologous proteins sharing the same CT domains have been identified in *A. baumannii*. The growth inhibition property of the two *A. baylyi* CdiA proteins was supported by competition assays between wild-type cells and mutants lacking immunity genes. However, neither protein plays a role in biofilm formation or adherence to epithelial cells, as proved by assays carried out with knockout mutants. Inhibitory and stimulatory properties may be similarly uncoupled in *A. baumannii* proteins.

**Electronic supplementary material:**

The online version of this article (10.1007/s00284-018-1540-y) contains supplementary material, which is available to authorized users.

## Introduction

Gram-negative bacteria exploit six secretion systems (types I–VI) to secrete proteins outside the cell [8]. Most secretion machineries are complex structures which span both the inner membrane (IM) and the outer membrane (OM). In the type V secretion system, proteins cross the IM through the Sec system and subsequently the OM either alone, in the auto-transporter pathway, or assisted by dedicated proteins, in the two-partner secretion (TPS) pathway [17]. The secreted proteins may remain onto the OM, be released into the extracellular milieu, or be injected into target cells. Secreted proteins are involved in different processes which include adhesion to cells or abiotic surfaces, iron acquisition, invasion of eukaryotic cells, environmental adaptation. Many are weapons used to prey upon non-self-bacteria. In type VI secretion systems, proteins evolutionarily related to phage tails components assemble contractile tubules which deliver killing proteins to adjacent cells [4].

In the peculiar TPS systems known as contact-dependent growth inhibition (CDI), CdiA proteins are exported on the outer membrane by cognate CdiB proteins and stop upon contact the growth of neighbouring bacteria [1, 16]. The C-terminal domain of CdiA (CdiA-CT) proteins contains a toxin activity delivered to targeted cells to inhibit their growth. Immunity CDI proteins neutralize toxin activity in CDI + bacteria. CDI systems had been identified in several Gram-negative bacteria, and multiple CdiA/CdiI toxin/antitoxin systems had been described in the same bacterial species [16, 20]. CdiA proteins promote also cooperative interactions between isogenic CDI + cells, facilitating biofilm formation. CDI-dependent cell–cell adhesion had been observed in *Escherichia coli* [1], *Xylella fastidiosa* [14], *Xanthomonas axonopodis* [13] *Burkholderia thailandensis* [12], *P. aeruginosa* [20]. The same holds for the type IV secretion system (T6SS), which has been shown to play an active role in kin recognition and territorial behaviour by exporting self-recognition proteins in *Proteus mirabilis* [28].

*Acinetobacter baylyi* is an environmental non-pathogenic bacterium occasionally found responsible for opportunistic infections [6], amenable to genetic modifications because highly competent in natural transformation [11]. In recent years, *A. baylyi* had been extensively analysed because provided of an efficient T6SS [23, 27] able to kill neighbouring bacteria and promote the acquisition of DNA from targeted cells [7].

The analysis of the complete genome sequence [3] revealed that the *A. baylyi* ADP1 strain potentially encodes two large surface proteins of 2000 and 3711 amino acids (orfs 2784 and 940, respectively), which were annotated as filamentous hemagglutinins.

The objectives of the present report were to (i) analyse the structural organization of these proteins and their relatedness to homologous proteins present in *A. baumannii*; (ii) demonstrate that they are components of two distinct CDI systems; (iii) analyse their role in biofilm formation and adherence to human pneumocytes.

## Materials and Methods

### Construction of *A. baylyi* Mutants

*Acinetobacter baylyi* ADP1 mutant strains were constructed by insertion of the kanamycin-resistance cassette into target genes as described previously [2]. Briefly, the *kanR* gene was PCR amplified from plasmid pCR2.1-TOPO (Thermo Fisher Scientific) using the KmFw and KmRv primers, and the upstream and the downstream regions of each target gene were amplified from ADP1 DNA using specific primer pairs. A nested overlap PCR was carried out with an Expand High Fidelity Taq DNA polymerase (Roche), using NestFw and NestRv primers to generate DNA fragments including the *kanR* cassette flanked by 400–700 bp of chromosomal regions upstream and downstream of the gene segment to be deleted. The nested overlap PCR was performed as previously described [9]. Transformation assays were performed as previously described [21]. Knockout deletions were verified in PCR experiments using primers CF and CR. Oligonucleotide primers are listed in Table S1. For biofilm assays, bacteria were grown in LB at 30 or 37 °C, or in brain heart infusion broth at 30 °C.

### Competition Assays

Overnight cultures were diluted in modified LB (10 g/l tryptone, 5 g/l yeast extract, 0.5 g/l NaCl) and incubated at 30 °C, till an A_600_ of ~1.0 was reached. Cultures were then diluted to an A_600_ of 0.4. For competition assays, 40 µl of predator *A. baylyi* ADP1 were mixed with 4 µl of prey cells and 20 µl of the mixture were spotted on LB-agar plates. Plates were incubated at 30 °C for 4 h. Then, spots were excised from the plate, placed in 500 µl of PBS, serially diluted, and plated on LB agar containing 12.5 µg/ml kanamycin. Of each dilution, 100 µl was spread for CFU count; 10 µl was spotted to visualize the outcome of the competition assay. Experiments were performed in triplicate.

### RNA Analyses

RNA was isolated from *A. baylyi* ADP1 cells at log, late-log, and stationary phases. To monitor expression levels of *cdiA* genes, RT-PCR analyses were carried out as previously described [24] using the oligonucleotides listed in Table S1. Transcript levels were normalized to 16S rRNA levels. Changes in transcript levels were determined by the relative quantitative method (ΔΔCT). Experiments were carried out in triplicate.

### Biofilm Formation Assay

Quantitative biofilm formation on polystyrene surfaces was investigated as previously reported [29]. To quantify biofilm formation in glass tubes, cultures were grown overnight in 12-mm-diameter glass under static conditions for 72 h at 30 °C. Upon medium removal, tubes were washed three times with PBS. Subsequently, 5 ml of 0.1% crystal violet was added for 15 min at room temperature, followed by rinsing with PBS. Tubes were photographed after air drying. For confocal laser scanning microscopy (CLSM) analyses, ~2 × 10^5^ CFU/ml of wild-type and mutant cells were added to cell culture plates containing glass coverslips and incubated in static conditions at 30 °C for 72 h. Biofilm images were recorded as previously described [9]. All experiments were performed in triplicate.

### Cell Adhesion Assays

Adherence of *A. baylyi* strains to A549 cells (human type 2 pneumocytes) was determined as described previously [9], with minor modifications. In brief, ~10^5^ A549 cells were infected with ~10^7^ bacterial CFU and incubated for 60 min at 37 °C in 5% CO_2_ (v/v) atmosphere. After removal of non-adherent bacterial cells by washing with PBS, infected cells were lysed by the addition of 1 ml distilled water and serial 10-fold dilutions were plated on LB agar to determine the number of CFU of adherent bacteria. Dilutions from harvested samples were seeded on LB agar plates and bacterial colony counts were assessed after overnight incubation at 37 °C. Each experiment was performed in triplicate.

### Statistical Analysis

Data were analysed using GraphPad Prism Version 5. Differences between mean values were tested for significance by performing one-way ANOVA analysis followed by Dunnett’s comparison test. A *P* value < 0.001 was considered to be statistically significant.

### In Silico Analyses

The CT regions of the CdiA2784 and CdiA940 proteins were used as queries for homology searches at GenBank carried out against both complete and draft genomes databases classified as *Acinetobacter* (taxid:469). Protein domains were searched at the NCBI CDD (Conserved Domain Database) site (http://www.ncbi.nlm.nih.gov/Structure/cdd/cdd.shtml). Protein alignments were generated with the CLUSTAL OMEGA program (https://www.ebi.ac.uk/Tools/msa/clustalo/). The sequence types of CDI^+^ strains were determined by querying either the genomes, or the pool of contig sequences of the strain of interest in FASTA format, against the *A. baumannii* MLST database (https://pubmlst.org/bigsdb?db=pubmlst_abaumannii_pasteur_seqdef&set_id=2&page=sequence).

## Results and Discussion

### *Acinetobacter baylyi* CDI Systems

**Fig. 1:**
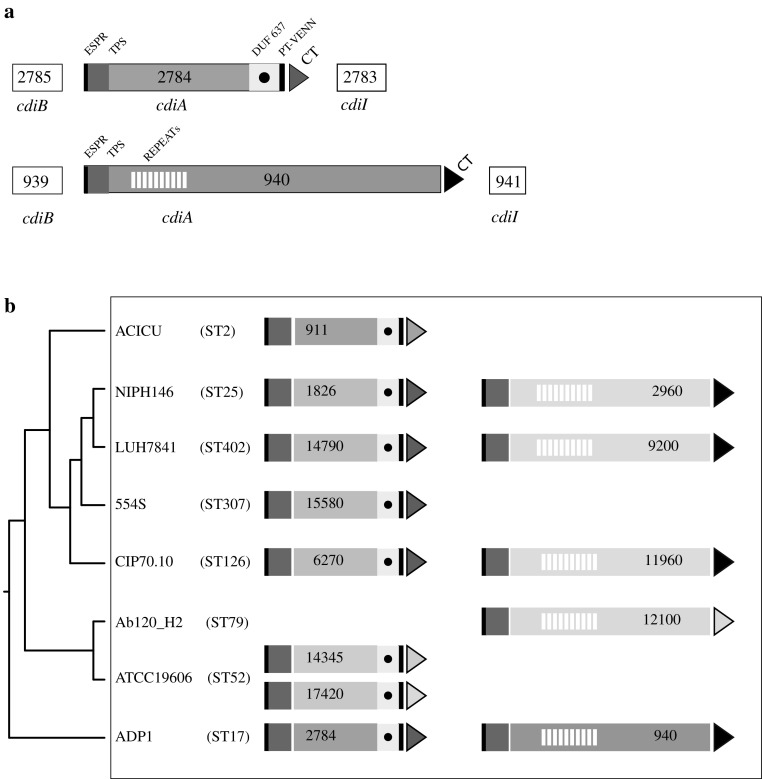
*Acinetobacter baylyi cdi* genes. **a** The two CDI gene sets found in the ADP1 strain are sketched. Numbers refer to the orfs corresponding to CdiB, CdiA, and CdiI. *ESPR* extended signal peptide region, *TPS* CdiB transporter binding domain, *DUF637* domain of unknown function 637, *PT VENN*, pre-toxin VENN domain, *CT* C-terminal toxic domain. **b** Representative *A. baumannii* strains featuring proteins homologous to *A. baylyi* CdiA are shown. For each, the ST (sequence type) is given in parenthesis. The cladogram results from the clustalW alignment of concatenated allele sequences of the *cpn60, fus, gltA, pyrG, recA, rplB*, and *rpoB* gene segments from the indicated ST. GenBank strain references: ACICU (CP000865), NIPH146 (NZ_KB849308), LUH_7841 (orf 14790, JZBX01000012.1; orf 9200, JZBX01000006.1), 554S (NKXP01000136.1), CIP70.10 (NZ_LN865143.1), AbH120-A2 (NZ_CP009534.1), ATCC19606 (NZ_KB849990.1). Proteins from ATCC19606 and AbH120-A2 have been described [14, 21]. Genes and coding sequences were not drawn to scale. Homologous CT domains have the same colour. (Color figure online)

The *A. baylyi* ADP1 strain features two *cdi* gene clusters. Each includes three adjacent genes, which encode the CdiB transporter, the secreted CdiA protein and an immunity CdiI protein, respectively (Fig. 1a). RT-PCR analyses revealed that both *cdi* gene clusters are transcribed (Fig. S1). The immunity genes *cdi*I941 and *cdi*I2783 are located at a distance of 1 and 12 bp from the upstream *cdi*A genes, respectively, and hence are co-transcribed with them. CdiA2784 and CdiA940 share an extended signal peptide region (ESPR) domain at the NH2 side, which is recognized by the Sec-translocation machinery and eventually cleaved during the export through the IM, and a TPS domain involved in CdiA–CdiB interactions, but differ in length and organization. CdiA2784 features a domain of unknown function (DUF637, PF04830), and a PT (pre-toxin)-VENN domain (PF04829), located, as in CdiA from many bacterial species [20], immediately upstream of the CT region. The CdiA2784 CT region spans a Tox-REase-7 domain (PF15649). CdiA940 features a large repeat region, constituted by arrays of short repeats reminiscent of those found in *(B) pertussis* filamentous hemagglutinin [18]. No toxin domain was recognized at the COOH terminus of CdiA940 at the NCBI Conserved Domain Database.

Homology searches showed that the two proteins were perfectly conserved in the few unfinished *A. baylyi* genomes deposited at GenBank (data not shown). CdiA2784 exhibits similarity to CdiA from *A. baumannii* reference strains ACICU and ATCC19606 (Fig. 1b), which carry different CT. Proteins exhibiting 62% identity to CdiA2784 and carrying similar CT regions (78% identity) were identified in several *A. baumannii* strains belonging to different lineages, including the epidemic sequence type (ST) 25 [26]. Some of these strains are shown in Fig. 1b. CdiA940 exhibited 41% identity to a large CdiA encoded by the *A. baumannii* Ab120-H2 strain [21], but 71% identity to large CdiA encoded by the same *A. baumannii* strains hosting CdiA2784 homologs. Noteworthy, these proteins feature the same CT region of CdiA940 (Fig. 1b). Sequence alignments of CdiA-CT regions are reported in figure S2. Data shown suggest that both *A. baylyi cdi*A genes may have been acquired from the same *A. baumannii* cell.

### Growth Competition Experiments

**Fig. 2:**
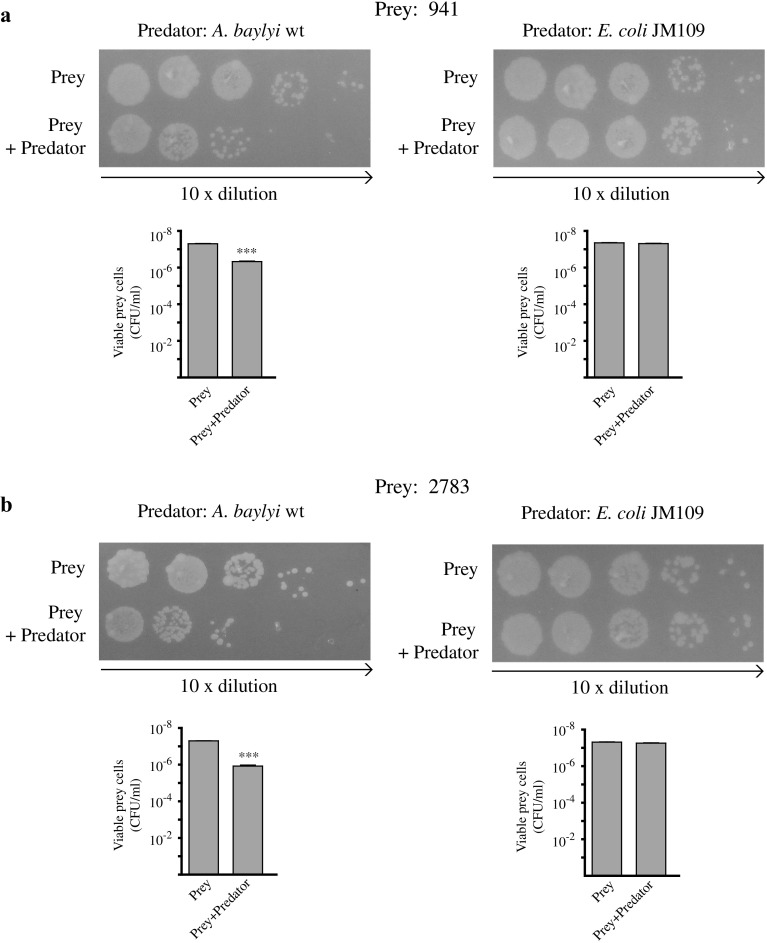
Growth competition assays. Δ941 (**a**) and Δ2783 (**b**) cells were incubated 4 h at 30 °C alone or mixed with a 10-fold excess of either *A. baylyi* ADP1 or *E. coli* JM109 cells before plating (see “Materials and Methods” for details). Images correspond to one representative experiment from three independent assays done with different cultures of prey and predator cells

CdiA proteins have been shown to have two opposite effects on neighbouring cells. They may inhibit the growth of non-self-bacteria, which lack immunity proteins antagonizing their toxins, but may also stimulate self-bacteria to build up biofilm structures. We asked whether the two different *A. baylyi* CdiA were able to perform both functions. *A. baylyi* cells should be self-protected against the toxic action of CdiA940 and CdiA2784 by the immunity proteins CdiI941 and CdiI2783 (Fig. 1). To validate the functioning of the two hypothesized toxin-antitoxin systems, we obtained deletion derivatives, in which the antitoxin *cdi*I941 and *cdi*I2783 genes were knocked out by the insertion of the kanamycin gene cassette [2]. The Δ941 and Δ2783 mutants were used as prey in competition experiments against tenfold excess of wild-type predator *A. baylyi* cells. As shown in Fig. 2, the growth of the antitoxin-minus Δ941 and Δ2783 cells was significantly inhibited by wild-type toxin-producers cells. For each experiment, the number of prey cells survived to competition was reported. Growth competition between mutant prey cells and non-toxic, mock predator *E. coli* cells were carried out as control, to rule out that 10-fold excess of predator cells, by reducing nutrients availability, may lower prey’s CFU in a non-specific way.

### Biofilm Formation and Adherence to A549 Human Bronchial Cells of *cdiA* Mutants

**Fig. 3:**
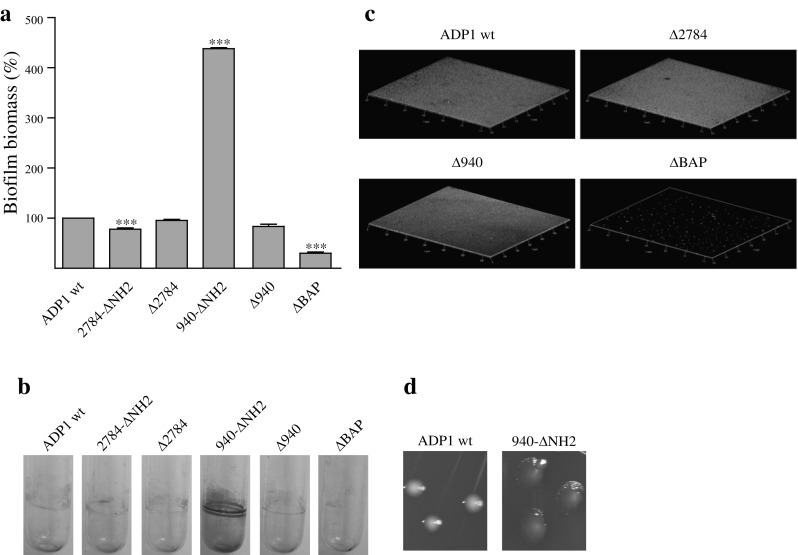
Biofilm formation of *A. baylyi* ADP1 cells, CdiA, and BAP mutants. **a** Quantitation of biofilm formation in 96 multi-well plates. Data are the means of three independent assays and presented as mean ± SEM, *P* < 0.001. Asterisks indicate statistically significant (*P* < 0.001) differences versus *A. baylyi* ADP1 strain. **b** Representative images of crystal violet (CV)-stained biofilms formed inside glass tubes. **c** Representative photographs of CLSM assays. Three-dimensional images are shown **d** Colonies formed by 940-ΔNH_2_ and wt *A. baylyi* ADP1 cells

Next, we asked whether CdiA2784 and CdiA940 enhance biofilm formation, similarly to CdiA in other bacterial species [1, 12–14, 20]. Two mutants of the *cdi*A940 gene were tested, in which DNA encoding residues 1–802 of CdiA940 (940-ΔNH_2_), or the entire *cdi*A gene (Δ940), was deleted. Biofilm formation was unaffected by the complete knockout of the *cdi*A940 gene but was surprisingly fourfold enhanced in 940-ΔNH_2_ cells, which resulted phenotypically different from wild-type cells (Fig. 3). Growth rates of wild-type and 940-ΔNH_2_ cells were identical, ruling out that changes in morphology and ability to form biofilm were correlated to increased number of 940-ΔNH_2_ cells.

Similar mutants of the *cdi*A2784 gene were obtained and assayed. The mutants 2784-ΔNH_2_ and Δ2784, in which DNA encoding residues 1–801 of CdiA2784 or the entire 2784 *cdi*A gene was deleted, respectively, produced biofilm with similar efficiency as parental *A. baylyi* ADP1 cells (Fig. 3). Altogether, data rule out that CdiA proteins play a role in biofilm formation. In 940-ΔNH_2_, an aberrant CdiA protein could be fortuitously translated and reach the OM, as shown for a *B. pertussis* hemagglutinin mutant lacking the TPS domain [10]. The increase in biofilm formation could have resulted from an altered surface presentation, due to either membrane misplacement or misfolding of the truncated CdiA940. The mutant was not further analysed.

In light of the results obtained, we knocked out also *A. baylyi* ADP1 orf 2866, which corresponds to the biofilm associated protein (*bap*) gene. BAP is a surface protein proved crucial for biofilm formation in *A. baumannii* [19]. BAP has similarly a significant role in the process of biofilm formation in *A. baylyi* (Fig. 3).

**Fig. 4:**
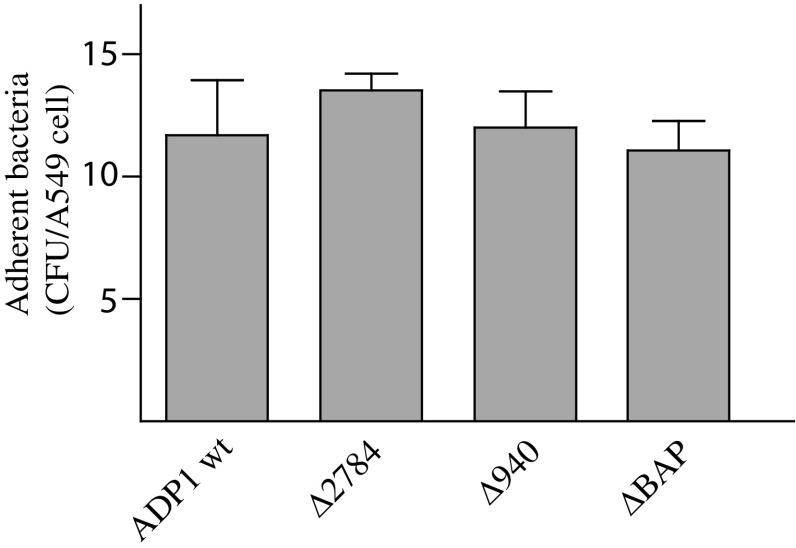
Adherence of *A. baylyi* ADP1 cells, CdiA and BAP mutants to A549 bronchial epithelial cells. The amount of cell surface-associated bacteria after 60-min incubation at 37 °C is shown. Asterisks denote statistically significant (*P* < 0.001) differences in the degree of cell adhesion

We monitored whether the loss of CdiA or BAP proteins could interfere with the ability of *A. baylyi* ADP1 cells to interact with A549 human bronchial cells. No significant differences were observed in the ability of CdiA and BAP mutants to adhere to A549 cells in comparison with wild-type *A. baylyi* ADP1 cells (Fig. 4). The length of the protruding structures formed by the *A. baylyi* (1725 aa) and the *A. baumannii (*8200 aa) BAP on the OM could plausibly explain why only the *A. baumannii* BAP stimulated bacterial adhesion to human epithelial cells [5].

## Conclusions

CdiA2784 and CdiA940 seem to function only as killing effectors since their absence does not impinge on the ability of *A. baylyi* cells to aggregate in biofilm structures. The finding that inhibitory and stimulatory properties are not associated in *A. baylyi* CdiA, as observed in most analogous proteins [1, 12–14, 20], is of interest, and foreseeable for the properties of *A. baumannii* CDI systems. Data are in agreement with a previous study [22], showing that the AbfhaB CdiA expressed by the *A. baumannii* AbH120-A2 strain (Fig. 1b) was crucial for adhesiveness to A549 cells but had no role in biofilm formation. The adhesiveness to A549 cells may reflect intrinsic differences between AbfhaB and CdiA940, or the presence of auxiliary factors enhancing the AbfhaB-dependent adhesion to eukaryotic cells of AbH120-A2.

The reason why BAP, but not CdiA proteins, stimulates biofilm formation is not known and merits further investigation. In *A. baumannii*, biofilm formation process is associated to the selective expression and regulation of a myriad of genes [25]. Taking into account that *cdi* are accessory genes, while *bap* genes are conserved component of *Acinetobacter* genomes [9], it is tempting to speculate that BAP proteins may be co-regulated with other proteins involved in biofilm formation in *Acinetobacter*.

## Electronic supplementary material

Below is the link to the electronic supplementary material.

Supplementary material 1 (PDF 72 KB)

Supplementary material 2 (PDF 353 KB)

Supplementary material 3 (PDF 59 KB)
