# Supplementary material for: Contact-Dependent Growth Inhibition Proteins in Acinetobacter baylyi ADP1
Source: Curr Microbiol. 2018 Jul 17;75(11):1434–40. doi: 10.1007/s00284-018-1540-y (PMC6182759; doi:10.1007/s00284-018-1540-y)
Supplement: Supplementary file 1 — Supplementary material 1 (PDF 72 KB) [file 284_2018_1540_MOESM1_ESM.pdf]

Table S1: Oligonucleotide primers used in the study.

Primer name	Sequence (5' to 3')	Application
Up Fw 940-ΔNH2	CTGTAGCAGGTGCCAGTCAA	Construction of the 940-ΔNH2 mutant
Up Rv 940-ΔNH2	CCCAGCTGGCAATTCCGGTGAAAATCTTGCTCAAACGATTC	
Dw Fw 940-ΔNH2	CTTGACGAGTTCTTCTGAATTGACATTACGGCAGAGCAA	
Dw Rv 940-ΔNH2	TATTCAGGCTACCCGCTTTG	
Nest Fw 940-ΔNH2	TTTAAGCGGCAATTTGACCTAC	
Nest Rv 940-ΔNH2	GCTTTGAGTTGCAAGGTTCC	
C Fw 940-ΔNH2	GAATCGTTTGAGCAAGATTTTCA	
C-wt Rv 940-ΔNH2	TGACCATCTGCCTGACTTTG	
C-del Rv 940-ΔNH2	TTGCTCTGCCGTAATGTCAA	
Up Fw Δ941	GTGCTGAGAATTTAGCAAAAGC	Construction of the Δ941 mutant
Up Rv Δ941	CCCAGCTGGCAATTCCGGTCATGCTGCTGTCATTGAATTTG	
Dw Fw Δ941	CTTGACGAGTTCTTCTGAATGGGTGGATAAGGTAGATTAGGA	
Dw Rv Δ941	CAGGCCCTTTAAAATCAACAA	
Nest Fw Δ941	CGTGGAATGGAATCACAAGA	
Nest Rv Δ941	TGCAATTTTGGCACCTGATA	
C Fw Δ941	GATTTCAGGGCCAGTACGTT	
C-wt Rv Δ941	CATCATTTTGGAAGCGATGA	
C-del Rv Δ941	AATCATGCGAAACGATCCTC	
Up Fw Δ940	CTGTAGCAGGTGCCAGTCAA	Construction of the Δ940 mutant
Up Rv Δ940	CCCAGCTGGCAATTCCGGTGAAAATCTTGCTCAAACGATTC	
Dw Fw Δ940	CTTGACGAGTTCTTCTGAATGGGTGGATAAGGTAGATTAGGA	
Dw Rv Δ940	CAGGCCCTTTAAAATCAACAA	
Nest Fw Δ940	TTTAAGCGGCAATTTGACCTAC	
Nest Rv Δ940	TGCAATTTTGGCACCTGATA	
C Fw Δ940	GAATCGTTTGAGCAAGATTTTCA	
C-wt Rv Δ940	TTGCTCTGCCGTAATGTCAA	
C-del Rv Δ940	CAGGTCGGTCTTGACAAAAAG	
Up Fw 2784-ΔNH2	TATCGGTGCCTTAGGACTCA	Construction of the 2784-ΔNH2 mutant
Up Rv 2784-ΔNH2	CCCAGCTGGCAATTCCGGAGTGGCTTAATAGCTGTAGTTCAAG	
Dw Fw 2784-ΔNH2	CTTGACGAGTTCTTCTGAAGCTCGAAGCAGGAGGTACAC	
Dw Rv 2784-ΔNH2	TGAGACAATGCTCCAAGTGC	
Nest Fw 2784-ΔNH2	AAGTCCAGCGCAGAATCACT	
Nest Rv 2784-ΔNH2	AGCATAATCCCCATCACCAA	
C Fw 2784-ΔNH2	CATGGGCAGTGTATTTGGTG	
C-wt Rv 2784-ΔNH2	CAGGAATGGATTGGCTCCTA	
C-del Rv2784-ΔNH2	GCCTCGTCTTGCAGTTCATT	
Up Fw Δ2783	GCTTTAATGGTCCGACTCCA	Construction of the Δ2783 mutant
Up Rv Δ2783	CCCAGCTGGCAATTCCGGTCAGGCCGCAGTATTTACGTCAT	
Dw Fw Δ2783	CTTGACGAGTTCTTCTGAATTTGACGGGTATTCTGAGGTC	
Dw Rv Δ2783	AGCCTGAAGTGCTGACACCT	
Nest Fw Δ2783	TGTTGTCGGTGCATTAGGAG	
Nest Rv Δ2783	CATTGGCTTCCCTGAGTTGT	
C Fw Δ2783	TGCTGGGGTAGGAGAAATTG	
C-wt Rv Δ2783	GGCTTTTTAGTTGCCGCATA	
C-del Rv Δ2783	TGTCTGTTGTGCCCAGTCAT	
Up Fw Δ2784	TATCGGTGCCTTAGGACTCA	Construction of the Δ2784 mutant
Up Rv Δ2784	CCCAGCTGGCAATTCCGGAGTGGCTTAATAGCTGTAGTTCAAG	
Dw Fw Δ2784	CTTGACGAGTTCTTCTGAATTAGAAAATCATAGGATTATTGTG	
Dw Rv Δ2784	TTTGGAACACTAAATACTCCCC	
Nest Fw Δ2784	AAGTCCAGCGCAGAATCACT	
Nest Rv Δ2784	TTTGGAACACTAAATACTCCCC	
C Fw Δ2784	CATGGGCAGTGTATTTGGTG	
C-wt Rv Δ2784	CAGGAATGGATTGGCTCCTA	
C-del Rv Δ2784	TATTTGTACGAATGGGTGTGCT	

Up Fw Δ2866	GGTGTGGTTTGTGGGTTAGG	Construction of the Δ2866 mutant
Up Rv Δ2866	CCCAGCTGGCAATTCCGGATGCACCCAATCCCTAACATT	
Dw Fw Δ2866	CTTGACGAGTTCTTCTGAAAGATGGCAGTGCTGGTCATA	
Dw Rv Δ2866	CCGCAAATCTGGCAATAACT	
Nest Fw Δ2866	GAAGATGCGTATGGCAATGA	
Nest Rv Δ2866	CTTCCACACAAGATGATGCAC	
C Fw Δ2866	AATGTTAGGGATTGGGTGCAT	
C-wt Rv Δ2866	TTGCTTGCATAGAGCAATGG	
C-del Rv Δ2866	TGTCTGTTGTGCCCAGTCAT	
KmFw	CCGGAATTGCCAGCTGGG	Kanamycin amplification
KmRv	TTCAGAAGAACTCGTCAAG	
RT Fw 940	GGTGCCATCCTCAATAATGC	RT-PCR for *cdiA*940 detection
RT Rv 940	TGGCTTCACCTTTGGCTAAC	
RT Fw 2784	TAGGAGCCAATCCATTCCTG	RT-PCR for *cdiA*2784 detection
RT Rv 2784	CCTGCAACTTCCAGATTTCC	
RT Fw 2866	ATTGGAGCAGGATCGCATAG	RT-PCR for *bap* detection
RT Rv 2866	AGTACGCTTTGGATCGATGG	
16S Fw	ATGGTCGGTACAAAGGGTTG	RT-PCR for 16S rRNA detection
16S Rv	TTCATGGAGTCGAGTTGCAG	
