# Supplementary material for: Contact-Dependent Growth Inhibition Proteins in Acinetobacter baylyi ADP1
Source: Curr Microbiol. 2018 Jul 17;75(11):1434–40. doi: 10.1007/s00284-018-1540-y (PMC6182759; doi:10.1007/s00284-018-1540-y)
Supplement: Supplementary file 2 — Supplementary material 2 (PDF 353 KB) [file 284_2018_1540_MOESM2_ESM.pdf]

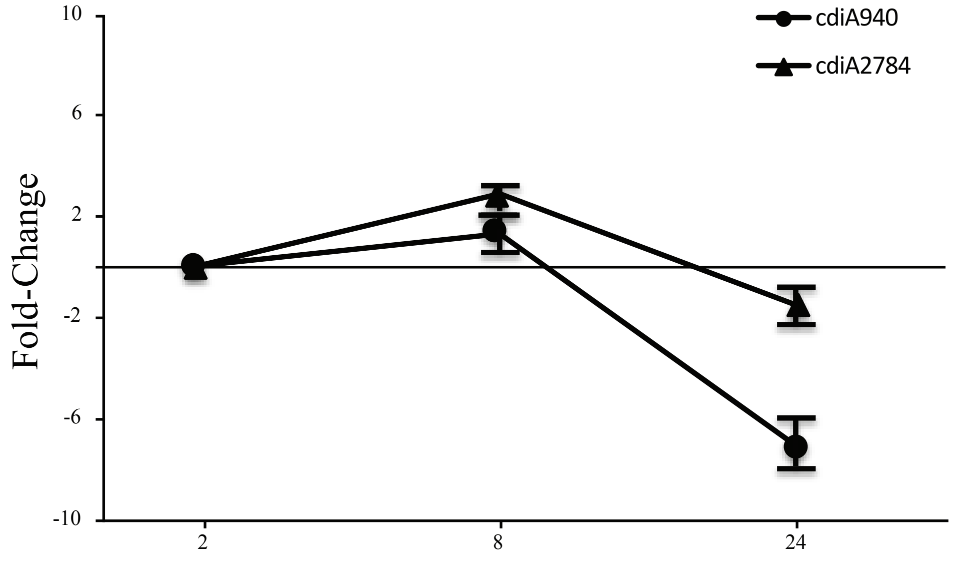

Figure S1. Changes of cdiA gene expression in A. baylyi ADP1 cells, grown at 30°C in LB broth, at 2, 8 and 24 hr as determined by qRT-PCR, using the relative quantitative method (ΔΔCT). Data shown are averages obtained from triplicate cultures. The error bars represent standard deviations.
