# Supplementary material for: Contact-Dependent Growth Inhibition Proteins in Acinetobacter baylyi ADP1
Source: Curr Microbiol. 2018 Jul 17;75(11):1434–40. doi: 10.1007/s00284-018-1540-y (PMC6182759; doi:10.1007/s00284-018-1540-y)
Supplement: Supplementary file 3 — Supplementary material 3 (PDF 59 KB) [file 284_2018_1540_MOESM3_ESM.pdf]

Supplementary file S2. Alignment of the CT regions of CdiA2784 (panel A) and CdiA940 (panel B) to homologous regions found in *A. baumannii* proteins. Dots denote identities.

A CdiA2784

```
ADP1        YAGYDVNTAANSADIAIQNN  SLVKLATSTGKILVKTLDEFNALRKAGKQVTKDDLVTSFKKQGADELIGIADDLLTVFGRSSSSFDRALAAIDLIVGTDLKPGKGESLKIAKNELEKLKTDRSYVQTVYQNKID
554S        YTGYDVNTAANSADTAIQNN  ...........V.......Y................A.L.E.S..................T......M.....V..............L..S...................V.
NIPH146     YTGYDVNTAANSADTAIQNN  ...........V.......Y................A.L.E.S..................T......M.....V..............L..S...................V.
LUH7841     YTGYDVNTAANSADTAIQNN  ...........V.......Y................A.L.E.S..................T......M.....V..............L..S...................V.
CIP70.10    YTGYDVNTAANSADTAIQNN  ...........V.......Y................A.L.E.S..................T......M.....V..............L..S...................V.
                PT-VENN module

ADP1 2784   VITRNRLNGKEFETSAASKLGIQRNTDRQMITVKTERDGQINIIPDAFGNGGTLVEFKNLKYITDTKQFRGYAATKKPVTLVINPDTKYSSTIENTIRDSKGIIYTFDQTTQKLKVLKDFRKS
554SV       –V....M........V.....VK.........I..........................VQ.L.....L......G...S..V.MN..I.E.VK.N.LSN..T.QR..PIK.T.HPY
NIPH146     .V....M........V.....VK.........I..........................VQ.L.....L......G...S..V.MN..I.E.VK.N.LSN..T.QR..PIK.T.HPY
LUH7841     .V....M........V.....VK.........I..........................VQ.L.....L......G...S..V.MN..I.E.VK.N.LSN..T.QR..PIK.T.HPY
CIP70.10    .V....M........V.....VK.........I..........................VQ.L.....L......G...S..V.MN..I.E.VK.N.LSN..T.QR..PIK.T.HPY
```

B CdiA940

```
ADP1        ASLKFANEVQKD  KQGAEYLINAASGIIVGGIAKGTVKNNNSDIDISKSGFEIGNINPITGHKILGTHKDESNNTVGNYIVEGTKPSTKDKVIKTDIQ
NIPH146     NYESLSNELSSS  ...........A..V....G..A..............................................................
CIP70.10    NYESLSNELSSS  ...........A..V....G..A..............................................................
LUH7841     NYESLSNELSSS  ...........A..V....G..A..............................................................

            YYENPGHHDPKGGNQIGYNKTKSVLPDNHKNLWDNSIVASDGNRWAVEVINGKKVYHRFQNDGNGNFHWNGSTNGKTASGVDRSDVSKIPPEILRK
            ..............................T.......................................................G.........
            ..............................T.......................................................G.........
            ..............................T.......................................................G.........
```
